# Supplementary material for: Haplotype-phased assemblies of the two poplar rust fungi species: Melampsora larici-populina and Melampsora allii-populina
Source: G3 (Bethesda). 2026 Aug 29;16(11):jkag247. doi: 10.1093/g3journal/jkag247 (PMC13732487; doi:10.1093/g3journal/jkag247)
Supplement: Supplementary file 1 [file jkag247_supplementary_data.zip › Supplementary_Figures_G3-2026-407029.pdf]

SUPPLEMENTARY FIGURES

Haplotype-phased assemblies of the two poplar rust fungi species: Melampsora larici-populina and Melampsora allii-populina

Corre et al.

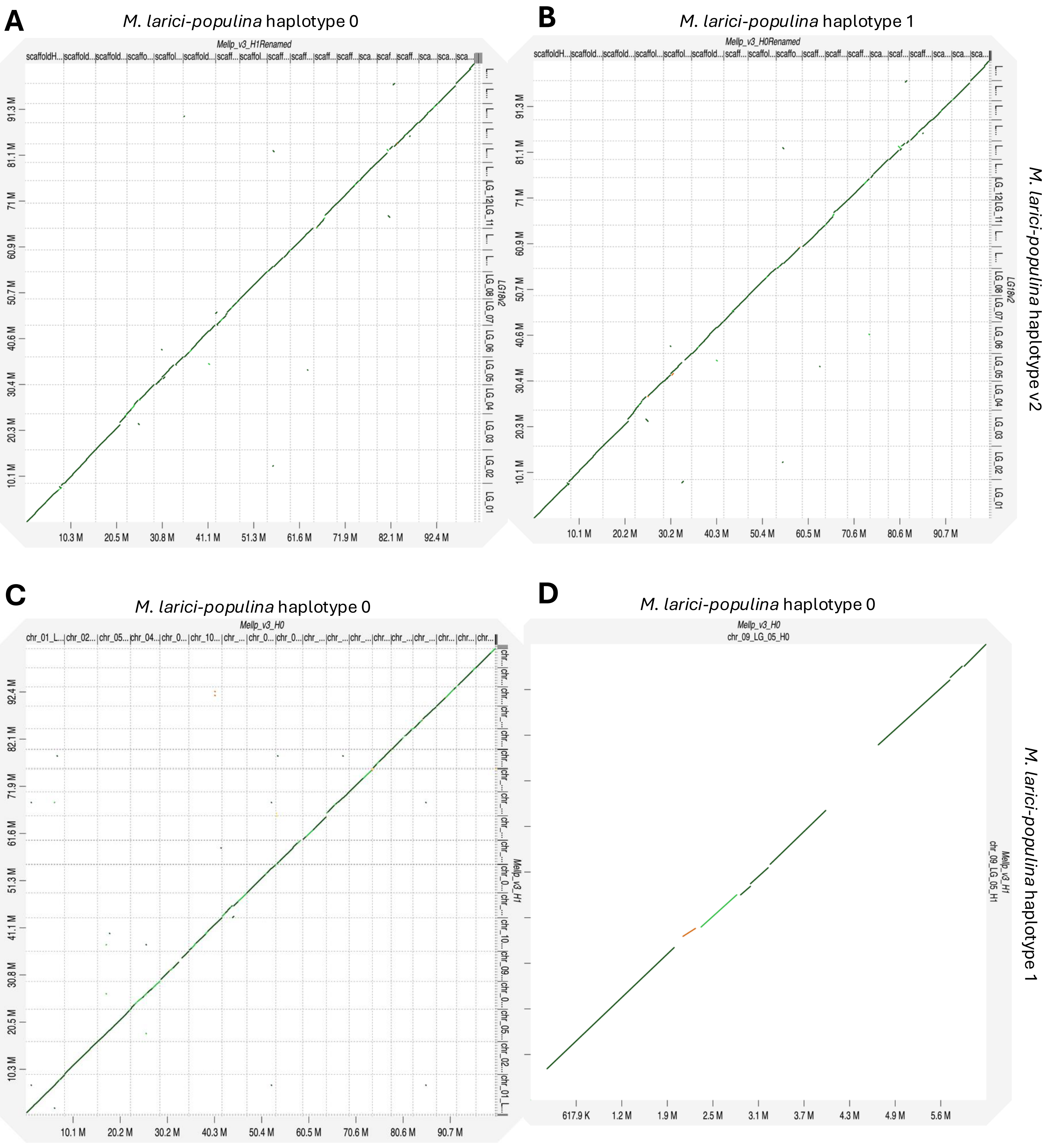

Supplementary Figure S1. Whole-genome dot plots of *Melampsora larici-populina* 98AG31 haplotype-phased assemblies and version 2 comparisons. A) Synteny dot plot of the newly assembled haplotype 0 (x-axis) against the previous reference genome version of *M. larici-populina* v2 (y-axis). B) Synteny dot plot of the newly assembled haplotype 1 (x-axis) against the *M. larici-populina* v2 (y-axis). C) Synteny dot plot between haplotype 0 (x-axis) and haplotype 1 of *M. larici-populina* (y-axis), showing high collinearity with limited structural variation. D) Close-up view of scaffold 9 synteny dot plot between haplotypes 0 (x-axis) and 1 (y-axis), highlighting a ~1.5-1.7 Mb non-syntenic region. This region corresponds to the mating-type locus containing the pheromone receptor genes, previously characterized in *M. lini* (Sperschneider et al., 2025).

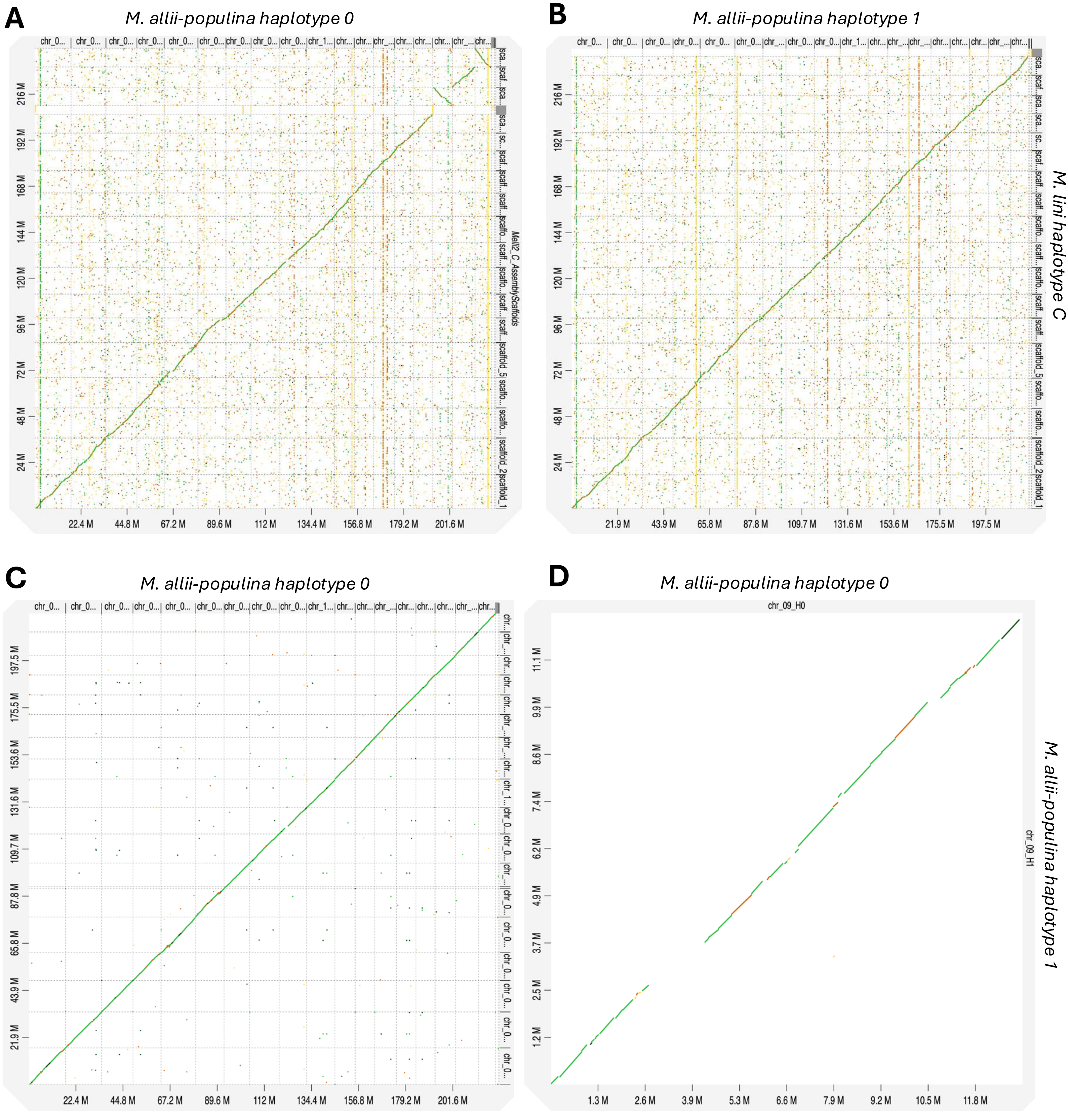

Supplementary Figure S2. Whole-genome dot plots of *Melampsora allii-populina* 12AY07 haplotype-phased assemblies and *Melampsora lini* C comparisons. A) Synteny dot plot of the *M. allii-populina* haplotype 0 (x-axis) against the previous reference genome version of *M. lini* C (y-axis). B) Synteny dot plot of the *M. allii-populina* haplotype 1 (x-axis) against the M. lini C (y-axis). C) Synteny dot plot between *M. allii-populina* haplotype 0 (x-axis) and *M. allii-populina* haplotype 1 (y-axis), showing high collinearity with limited structural variation. D) Close-up view of scaffold 9 synteny dot plot between *M. allii-populina* haplotypes 0 (x-axis) and 1 (y-axis), highlighting a ~2 Mb non-syntenic region. This region corresponds to the mating-type locus containing the pheromone receptor genes, previously characterized in *M. lini* (Sperschneider et al. 2025).

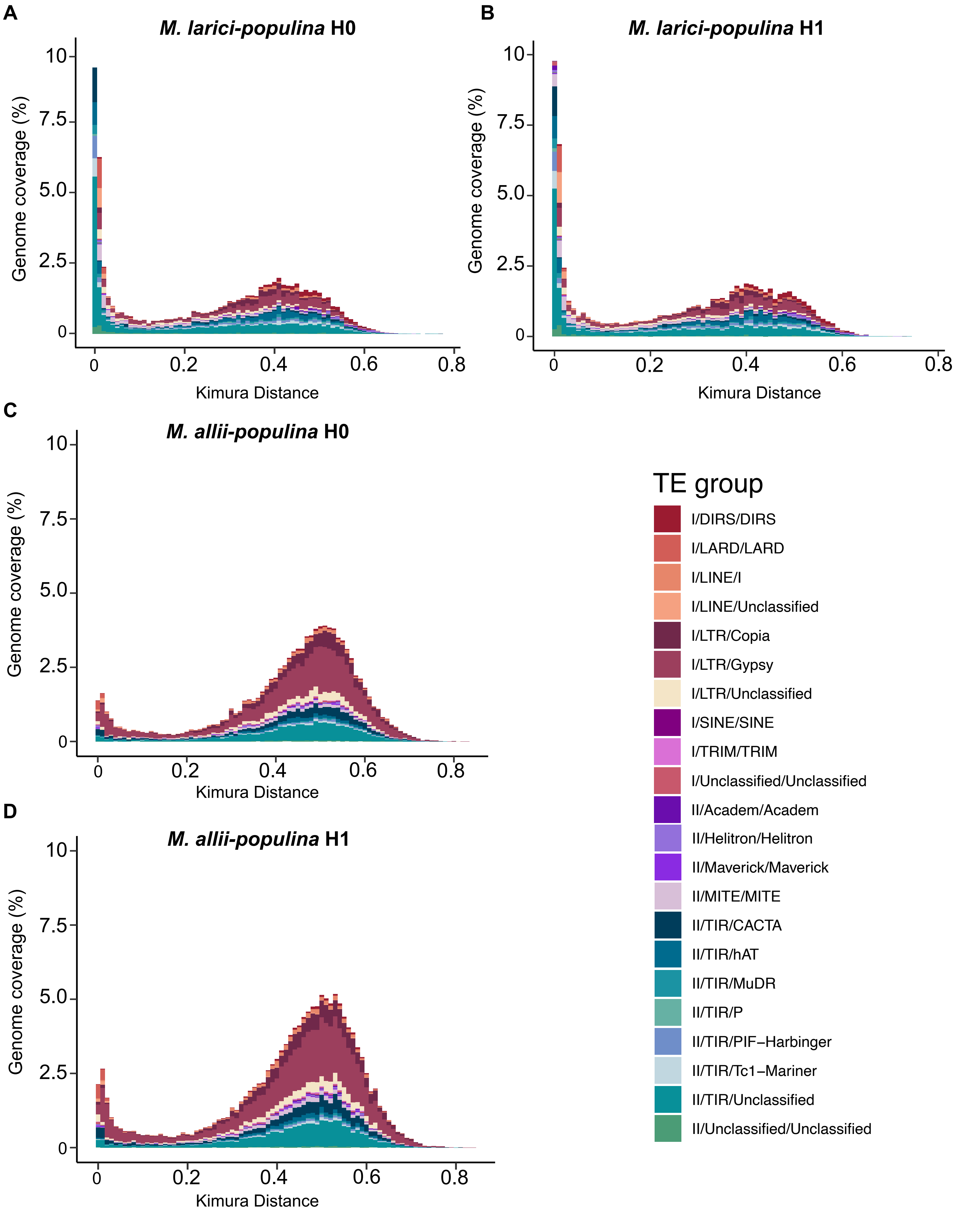

Supplementary Figure S3: Transposable element insertion ages in the two poplar-rust species. Kimura distances across the TE landscapes of the genomes of A) *M. larici-populina* haplotype 0, B) *M. larici-populina* haplotype 1, C) *M. allii-populina* haplotype 0, D) *M. allii-populina* haplotype 1. Each distribution shows the proportion of the genome (y-axis) composed of copies exhibiting varying degrees of divergence (x-axis).
