# Supplementary material for: Haplotype-phased assemblies of the two poplar rust fungi species: Melampsora larici-populina and Melampsora allii-populina
Source: G3 (Bethesda). 2026 Aug 29;16(11):jkag247. doi: 10.1093/g3journal/jkag247 (PMC13732487; doi:10.1093/g3journal/jkag247)
Supplement: Supplementary file 1 [file jkag247_supplementary_data.zip › Supplementary_Material_Legends_G3-2026-407029.docx]

**SUPPLEMENTARY MATERIAL LEGENDS**

**Supplementary Figure S1. Whole-genome dot plots of *Melampsora larici-populina* 98AG31 haplotype-phased assemblies and version 2 comparisons.** A) Synteny dot plot of the newly assembled haplotype 0 (x-axis) against the previous reference genome version of *M. larici-populina* v2 (y-axis). B) Synteny dot plot of the newly assembled haplotype 1 (x-axis) against the *M. larici-populina* v2 (y-axis). C) Synteny dot plot between haplotype 0 (x-axis) and haplotype 1 of *M. larici-populina* (y-axis), showing high collinearity with limited structural variation. C) Close-up view of scaffold 9 synteny dot plot between haplotypes 0 (x-axis) and 1 (y-axis), highlighting a ~1.5-1.7 Mb non-syntenic region. This region corresponds to the mating-type locus containing the pheromone receptor genes, previously characterized in *M. lini*.

**Supplementary Figure S2. Whole-genome dot plots of *Melampsora allii-populina* 12AY07 haplotype-phased assemblies and *Melampsora lini C* comparisons.** A) Synteny dot plot of the *M. allii-populina* haplotype 0 (x-axis) against the previous reference genome version of *M. lini C* (y-axis). B) Synteny dot plot of the *M. allii-populina* haplotype 1 (x-axis) against the *M. lini C* (y-axis). C) Synteny dot plot between *M. allii-populina* haplotype 0 (x-axis) and *M. allii-populina* haplotype 1 (y-axis), showing high collinearity with limited structural variation. C) Close-up view of scaffold 9 synteny dot plot between *M. allii-populina* haplotypes 0 (x-axis) and 1 (y-axis), highlighting a ~2 Mb non-syntenic region. This region corresponds to the mating-type locus containing the pheromone receptor genes, previously characterized in *M. lini*.

**Supplementary Figure S3. Transposable element insertion ages in the two poplar-rust species.** Kimura distances across the TE landscapes of the genomes of A) *M. larici-populina* haplotype 0, B) *M. larici-populina* haplotype 1, C) *M. allii-populina* haplotype 0, D) *M. allii-populina* haplotype 1. Each distribution shows the proportion of the genome (y-axis) composed of copies exhibiting varying degrees of divergence (x-axis).

**Supplementary Table S1. Putative centromeric regions annotation based on Hi-C contact maps.**

**Supplementary Table S2.** **Intra- versus inter-haplotype Hi-C contact statistics supporting the haplotype phasing of Melampsora larici-populina v3.0.** For each contig, the percentage of Hi-C contacts to haplotype 0 and to haplotype 1 is shown, with the total Hi-C contact frequency and contig length. Contigs were assigned to haplotypes with NuclearPhaser (Duan et al., 2022). Values were computed at the contig level, prior to final scaffolding. Only contigs corresponding to the 18 chromosome-scale scaffolds per haplotype are shown.

**Supplementary Table S3.** **Intra- versus inter-haplotype Hi-C contact statistics supporting the haplotype phasing of Melampsora allii-populina v2.0.** For each contig, the percentage of Hi-C contacts to haplotype 0 and to haplotype 1 is shown, with the total Hi-C contact frequency and contig length. Contigs were assigned to haplotypes with NuclearPhaser (Duan et al., 2022). Values were computed at the contig level, prior to final scaffolding. Only contigs corresponding to the 18 chromosome-scale scaffolds per haplotype are shown.

**Supplementary Table S4. Mating-type genes BLAST search results of in *Melampsora larici-populina* v3.0 and *Melampsora allii-populina* v2.0.** MAT genes retrieved with blast against the genomes of *M. larici-populina 98AG31 haplotype 0, M. larici-populina 98AG31 haplotype 1, M. allii-populina haplotype 0, M. allii-populina haplotype 1, M. lini haplotype C, M. lini haplotype H.*

**Supplementary Table S5. Transposable elements annotations in *Melampsora larici-populina* at the Class, Order and superfamily levels**

**Supplementary Table S6. Transposable elements annotations in *Melampsora allii-populina* at the Class, Order and superfamily levels**

**Supplementary Table S7. Functional annotation summaries in *Melampsora larici-populina* (Mellp) and *Melampsora allii-populina* (Melap).**

**Supplementary Table S8. *Melampsora larici-populina* H0 gene functions.**

Protein functional annotations are summarized per predicted protein (proteinId) and linked to the corresponding gene (gene_id) and transcript (transcriptId) identifiers from the GFF3, together with the GFF3 Name field (gff_Name) and the predicted product description (gff_product). InterPro/domain annotations are reported as a concatenated list of individual hits (domain.domain_hits) and as aggregated fields listing the InterPro entry identifiers (domain.iprId) and descriptions (domain.iprDesc), as well as the source signature database (domain.domainDb) with its accession (domain.domainId) and description (domain.domainDesc). eggNOG-derived functional assignments include predicted enzyme commission numbers (eggnog.ec), KEGG orthology identifiers (eggnog.keggKo) and associated KEGG pathway/module/reaction information (eggnog.keggPathway, eggnog.keggModule, eggnog.keggReaction, eggnog.keggRclass), KEGG BRITE categories (eggnog.brite), and transporter classification codes when applicable (eggnog.keggTc), together with the supporting search statistics (eggnog.eValue, eggnog.score). Signal peptide predictions from SignalP are provided as the predicted class (signalp.prediction), the cleavage site interval (signalp.min_location, signalp.max_location) and the signal peptide probability (signalp.sp_prob).

**Supplementary Table S9.** ***Melampsora larici-populina* H1 gene functions.**

Protein functional annotations are summarized per predicted protein (proteinId) and linked to the corresponding gene (gene_id) and transcript (transcriptId) identifiers from the GFF3, together with the GFF3 Name field (gff_Name) and the predicted product description (gff_product). InterPro/domain annotations are reported as a concatenated list of individual hits (domain.domain_hits) and as aggregated fields listing the InterPro entry identifiers (domain.iprId) and descriptions (domain.iprDesc), as well as the source signature database (domain.domainDb) with its accession (domain.domainId) and description (domain.domainDesc). eggNOG-derived functional assignments include predicted enzyme commission numbers (eggnog.ec), KEGG orthology identifiers (eggnog.keggKo) and associated KEGG pathway/module/reaction information (eggnog.keggPathway, eggnog.keggModule, eggnog.keggReaction, eggnog.keggRclass), KEGG BRITE categories (eggnog.brite), and transporter classification codes when applicable (eggnog.keggTc), together with the supporting search statistics (eggnog.eValue, eggnog.score). Signal peptide predictions from SignalP are provided as the predicted class (signalp.prediction), the cleavage site interval (signalp.min_location, signalp.max_location) and the signal peptide probability (signalp.sp_prob).

**Supplementary Table S10.** ***Melampsora allii-populina* H0 gene functions.**

Protein functional annotations are summarized per predicted protein (proteinId) and linked to the corresponding gene (gene_id) and transcript (transcriptId) identifiers from the GFF3, together with the GFF3 Name field (gff_Name) and the predicted product description (gff_product). InterPro/domain annotations are reported as a concatenated list of individual hits (domain.domain_hits) and as aggregated fields listing the InterPro entry identifiers (domain.iprId) and descriptions (domain.iprDesc), as well as the source signature database (domain.domainDb) with its accession (domain.domainId) and description (domain.domainDesc). eggNOG-derived functional assignments include predicted enzyme commission numbers (eggnog.ec), KEGG orthology identifiers (eggnog.keggKo) and associated KEGG pathway/module/reaction information (eggnog.keggPathway, eggnog.keggModule, eggnog.keggReaction, eggnog.keggRclass), KEGG BRITE categories (eggnog.brite), and transporter classification codes when applicable (eggnog.keggTc), together with the supporting search statistics (eggnog.eValue, eggnog.score). Signal peptide predictions from SignalP are provided as the predicted class (signalp.prediction), the cleavage site interval (signalp.min_location, signalp.max_location) and the signal peptide probability (signalp.sp_prob).

**Supplementary Table S11.** ***Melampsora allii-populina* H1 gene functions.**

Protein functional annotations are summarized per predicted protein (proteinId) and linked to the corresponding gene (gene_id) and transcript (transcriptId) identifiers from the GFF3, together with the GFF3 Name field (gff_Name) and the predicted product description (gff_product). InterPro/domain annotations are reported as a concatenated list of individual hits (domain.domain_hits) and as aggregated fields listing the InterPro entry identifiers (domain.iprId) and descriptions (domain.iprDesc), as well as the source signature database (domain.domainDb) with its accession (domain.domainId) and description (domain.domainDesc). eggNOG-derived functional assignments include predicted enzyme commission numbers (eggnog.ec), KEGG orthology identifiers (eggnog.keggKo) and associated KEGG pathway/module/reaction information (eggnog.keggPathway, eggnog.keggModule, eggnog.keggReaction, eggnog.keggRclass), KEGG BRITE categories (eggnog.brite), and transporter classification codes when applicable (eggnog.keggTc), together with the supporting search statistics (eggnog.eValue, eggnog.score). Signal peptide predictions from SignalP are provided as the predicted class (signalp.prediction), the cleavage site interval (signalp.min_location, signalp.max_location) and the signal peptide probability (signalp.sp_prob).
